# Glenoid Augmentation with the Long Head Biceps for Shoulder Instability (GALIN)

**DOI:** 10.1016/j.xrrt.2026.100777

**Published:** 2026-05-15

**Authors:** Srinath Kamineni, Luis Angel Comas Marrero, Cristofer Muench, Jared Alexander Marsh

**Affiliations:** a Department of Orthopaedic Surgery, Indiana University School of Medicine, Evansville, IN, USA; b Orthopaedics & Sports Medicine, Wabash General Hospital, Mount Carmel, IL, USA; c Department of Orthopaedic Surgery and Sports Medicine, University of Kentucky College of Medicine, Lexington, KY, USA

**Keywords:** Glenoid labrum reconstruction, Shoulder instability surgery, Long head of biceps tendon (LHBT), Neo-labrum technique, Arthroscopic stabilization, Bankart and Latarjet alternatives, Glenoid bone loss, Labrum

The glenoid labrum is a circumferential fibrocartilaginous structure partially responsible for humeral head stability. The labrum deepens the glenoid fossa, thereby increasing the size of the glenoid track (the articulation between the humeral head and the glenoid bone), and hence improving joint stability, while maintaining range of motion.^13^

Disruption of the glenoid labrum, due to joint dislocation, results in the loss of joint constraint, thereby compromising shoulder stability.^15^ Traumatic shoulder joint subluxations and dislocations can cause detachment or abrasion of the glenoid labrum in addition to the joint capsule and ligaments. In order to stabilize the joint following traumatic shoulder instability, various surgical repair techniques have been developed, including both soft tissue and bony procedures. The Bankart repair (reattaching the torn glenoid labrum to its anatomic position) is the current gold standard for repairing anterior-inferior glenoid labrum tears (Bankart lesions).^22^ While the traditional open Bankart repair improves stability, the arthroscopic Bankart repair is associated with a higher redislocation risk in meta-analysis.^14^

Techniques other than the Bankart repair have been developed and employed with varying success. Many current methods of repair (notably glenohumeral ligament reattachment with capsular tightening) retain a significant risk of recurrent subluxation/dislocation events, indicated by the long-term results of the common capsular plication procedures, subscapular augmentations, and rotator interval narrowing procedures.^3^ In addition to failing to replicate the original structural stability afforded by the native labrum, the aforementioned procedures often result in reduced patient range of motion (commonly external rotation) and progressive early joint degenerative changes.^3^^,^^17^^,^^21^^,^^25^ In contemporary practice, arthroscopic Bankart repair is frequently combined with remplissage when clinically indicated, and short-term comparative data suggest that Bankart repair with remplissage can yield recurrence rates comparable to Latarjet in select patients.^12^ The dynamic anterior stabilization (DAS) technique aims to minimize the reduction of the patient's range of motion while simultaneously increasing shoulder stabilization. Although the DAS technique with Bankart repair displayed significant anterior stabilization in cadaveric studies, its efficacy was still underwhelming compared to the original Latarjet procedure.^18^ This leaves the patient with the difficult decision to prioritize increased shoulder stabilization for a decrease in range of motion and vice versa.

A novel technique, developed and in clinical use by the senior author for over 20 years, recreates a passive stabilizing labral “bumper” while aiming to preserve range of motion. Entitled “Glenoid Augmentation with the Long Head Biceps for Instability” (GALIN technique), this arthroscopically repurposes the ipsilateral long head of the biceps tendon (LHBT) to recreate a “neo-labrum” in lieu of a deficient glenoid labrum. Although other LHBT-based labral reconstruction/augmentation techniques have been described (including free autograft and onlay constructs), the GALIN technique uniquely uses an in-continuity proximal biceps graft combined with a distal (suprapectoral) tenodesis, allowing a local autograft without additional donor-site morbidity.^1^^,^^4^^,^^9^

## Methods/surgical technique

### Indications—all 4 are required to proceed with the technique

1. Recurrent anterior shoulder instability with substantial soft tissue deficiency (eg, attenuated or absent labrum and/or capsular insufficiency) in which primary capsulolabral repair is unlikely to provide durable stability.
2. Revision instability with poor-quality capsulolabral tissue, particularly in cases where prior stabilization has compromised or depleted viable labral tissue.
3. Structurally viable LHBT, confirmed to be intact and suitable for use.
4. Minimal glenoid bone loss; in carefully selected patients, mild subcritical glenoid bone loss (approximately ≤15%) may be acceptable when the predominant pathology is soft tissue insufficiency and the glenoid rim maintains adequate concavity.

### Contraindications/relative contraindications

1. Critical glenoid bone loss or a bipolar bone loss pattern is more appropriately treated with bony augmentation procedures (eg, Latarjet, free bone-block reconstruction).^5^
2. Poor candidate for arthroscopy or inability/unwillingness to adhere to prolonged post-operative immobilization and rehabilitation.
3. Active infection or concern for uncontrolled systemic inflammatory arthropathy.
4. Documented nonadherence/noncompliance with post-operative restrictions or follow-up.
5. Connective tissue disorders, including Ehlers–Danlos syndrome or other collagen vascular diseases.

### Assessing and preparing joint

**Figure 1:**
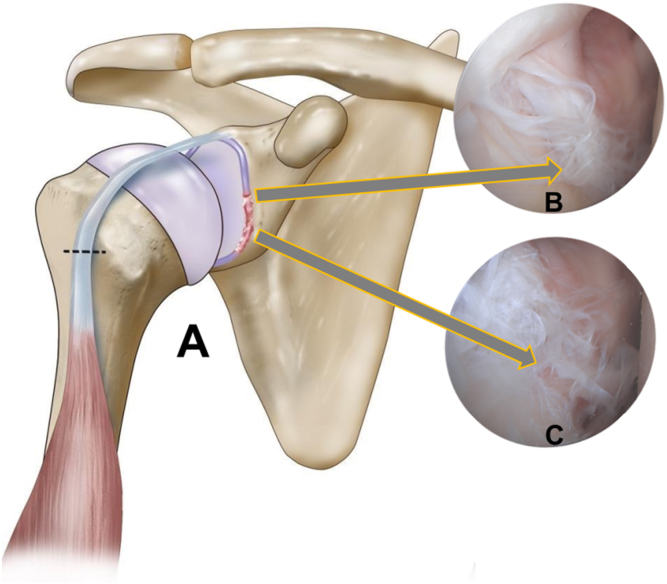
(**A**) Illustration of a deficient non-reconstructable anterior labrum. (**B**) Arthroscopic view of 12-3 o'clock anterior labral remnant, (**C**) Arthroscopic view of 3-5 o'clock labral remnant. Dashed line is the intended LHBT transection site. *LHBT*, long head of the biceps tendon.

The patient, under general anesthesia, is placed in the lateral decubitus position (modifiable to beach chair), and longitudinal traction is applied to the arm with a position of 30 degrees of flexion and abduction. Standard portals include a posterolateral viewing portal, anteroinferior portal (superior margin of subscapularis), anterosuperior portal (posterior margin of the rotator interval), and an accessory bicipital-groove portal. The accessory portal is discussed in further detail below. The posterolateral portal is used for a 30-degree arthroscope; the anteroinferior and accessory portals are working portals. We typically use cannulas in the working portals (eg, 8–8.25 mm in the anteroinferior portal and 5–6 mm in the accessory portal) to facilitate suture management and tendon handling. A 70-degree arthroscope may be helpful for more difficult pathologies or larger patients. Initially, a diagnostic arthroscopy is performed to assess the joint's soft tissue and bony structures and to visualize and probe the quality of the anterior/posterior static constraints, including the glenoid, its cartilage surface, the labrum, anteroinferior glenohumeral ligament complex, and the capsule (Fig. 1).

If glenoid bone loss is subcritical, repair using the LHBT may be considered in carefully selected patients with labral deficiency and with <15% glenoid bone loss, while also considering age, activity level, hyperlaxity, and revision status.^23^ In patients with >15% bone loss and/or significant bipolar bone loss, we preferentially consider a bony augmentation procedure (eg, Latarjet, iliac crest/distal tibial allograft) or alternative stabilization strategies; arthroscopic Bankart repair with remplissage may be an option in select cases based on shared decision-making and emerging comparative data.^12^

After diagnostic arthroscopy, the native capsulolabral complex is assessed before committing to reconstruction. The anterior nonarticular pathologic zone of the glenoid is débrided using a combination of arthroscopic soft tissue shavers and a radiofrequency probe for the removal of damaged and unreconstructable labrum. Only the intended healing surface is refreshed on the anterior glenoid, and the “scarred” labrum is not excised until its reconstructibility is determined. Bony preparation is performed to expose a bleeding surface at the intended fixation footprint. Using elevators, the residual labrum is carefully mobilized off the scarred anterior glenoid face/neck, typically from approximately the 2 o'clock to 6 o'clock positions. Following complete mobilization, tissue quality is reassessed. More specifically, the elasticity, thickness, and ability to accept and hold suture. When the mobilized labrum demonstrates adequate structural integrity, we preferentially perform a primary capsulolabral repair/advancement using native tissue, as we have encountered cases in which seemingly deficient labrum becomes repairable after mobilization. Only when the capsulolabral tissue remains attenuated, fragmented, or otherwise irreparable after full mobilization do we proceed with the GALIN technique.

### Tendon harvest and tenodesis of long head of the biceps tendon

**Figure 2:**
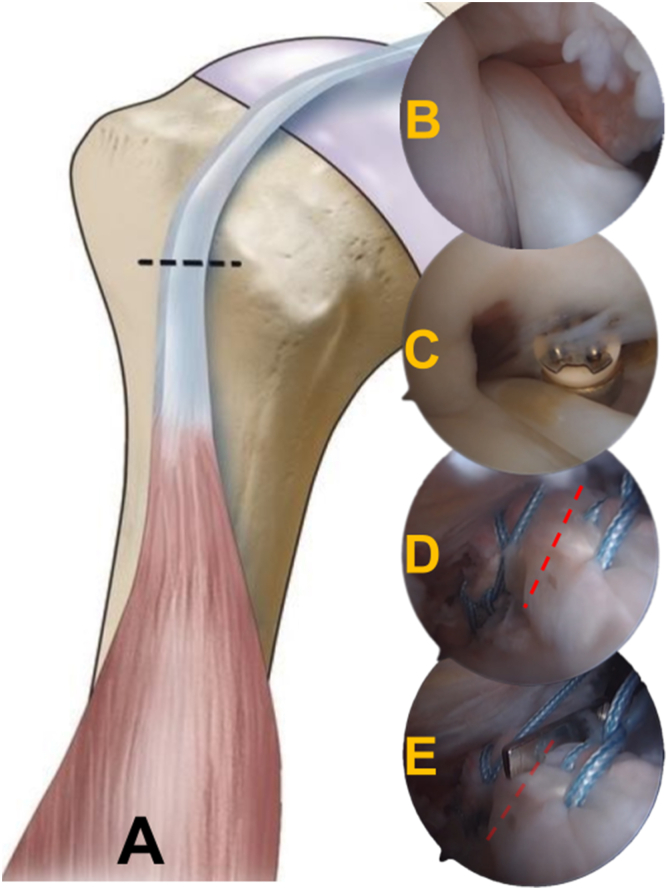
(**A**) Illustration of the LHBT tendon, marked at the site of intended transection (dotted line), proximal to which is the measured autograft for GALIN neo-labrum reconstruction, and distal to which is the LHBT tenodesis. (**B**) LHBT arthroscopically observed to exit the joint into the bicipital sulcus, (**C**) Débridement of surrounding synovitis with radiocautery, (**D**) Two Krackow stitches placed on either side of the transection site (dotted line), (**E**) Arthroscopic scissors used to transect the LHBT. *LHBT*, long head of the biceps tendon; *GALIN*, Glenoid Augmentation with the Long Head Biceps for Instability.

Once the extent of the static stabilizer deficiency is documented, the length of the required LHBT (neo-labrum) can be harvested. The arthroscope is removed from the posteroinferior viewing portal and reinserted into the anterosuperior portal. The LHBT is followed with the arthroscope into the bicipital sheath under the transverse bicipital ligament, and the ligament is transected to allow the necessary space for the arthroscope to progress distally (Fig. 2). The accessory working portal is created using an outside-in technique. With the arthroscope in the anterolateral portal, the bicipital groove is localized with finger palpation, and a spinal needle is introduced under direct visualization at the desired tenodesis level (suprapectoral, just distal to the transverse humeral ligament), approximately 1–2 cm distal to the humeral articular surface. A small skin incision is made, a blunt hemostat spreading technique is used to enter the bicipital groove (Fig. 2), and then a 6-mm cannula is inserted. While we do not traditionally use a cannula, one may be easily utilized. The musculotendinous junction of the LHBT and the planned transection site are identified and marked based on the required graft length, approximately 1–2 cm proximal to the musculotendinous junction. Five mm distal and 5 mm proximal ends of the transection site are captured using locking whipstitches (eg, Krackow), performed with an arthroscopic suture passer (Arthrex Scorpion) using #2 nonabsorbable FiberWire with 3–4 locking throws on each side and long tails for shuttling. Once both sutures are secured in multiple locking throws through the biceps tendon, the tendon is transected between the sutures with arthroscopic scissors. With the arm in neutral rotation and elbow flexed, taking care to avoid overtensioning, the distal captured suture is passed through the distal extent of the transverse humeral ligament multiple times and tied in place to create a soft tissue tenodesis of the biceps at physiologic length–tension. Fixation is confirmed by probing the tenodesis site before proceeding. The remaining suture of the distal stump is removed, and the proximal stump with the suture is passed into the joint, ready for the reconstruction portion of the procedure. However, it is feasible to tenodese into the humerus using an interference screw such as a SwiveLock/PushLock (Arthrex, Naples, FL, USA).

**Figure 3:**
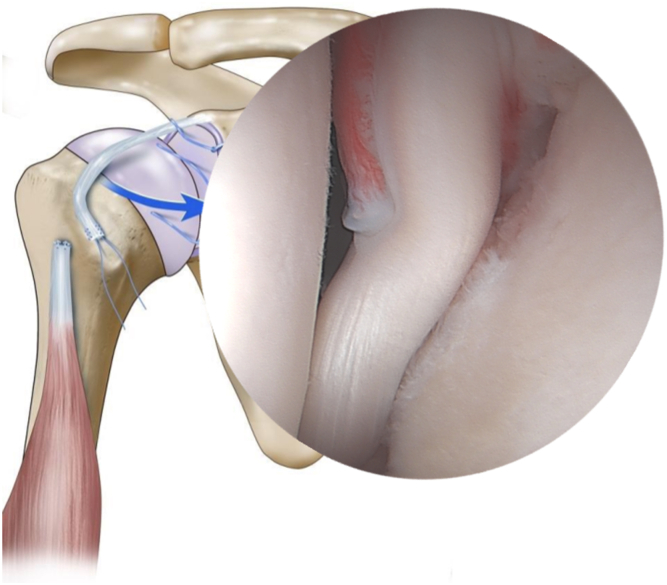
Using the knot pusher, the cut end of the graft is brought into the joint and transported toward the 6 o'clock position.

The arthroscope is then reinserted into the humeral joint space via the posterolateral portal. The proximal tendon remains attached at its superior labral origin. The suture that secured the proximal end of the tendon is passed into the joint with a knot pusher, and the suture is captured with a grasper through the anterolateral portal, delivering the tendon into the joint space. The knot pusher is used to push the cut end of the tendon graft toward the 6 o'clock position (Fig. 3).

### Attaching long head of the biceps tendon autograft (neo-labrum)

**Figure 4:**
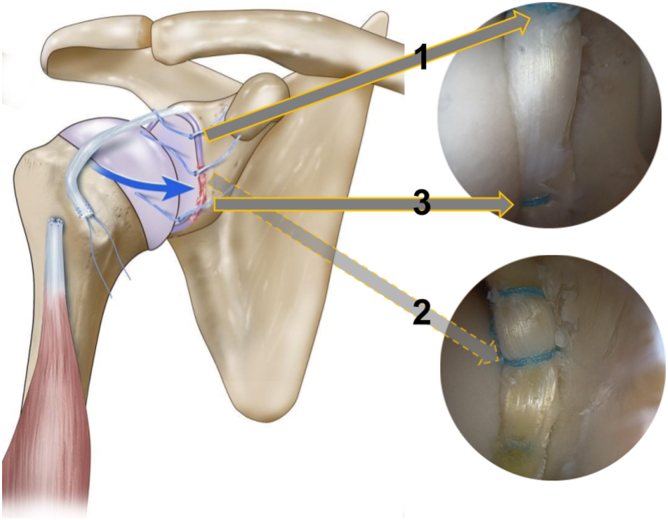
Tendon fixation sequence (right shoulder): (1) an anchor is set at the 1 o'clock position to capture the tendon near its origin; (2) an anchor is placed at the 5 o'clock position (often with a curved guide) to tension the anteroinferior glenohumeral ligament complex; (3) an anchor is placed at the 3 o'clock position to complete fixation and recreate a continuous bumper (left shoulder: 11, 7, and 9 o'clock).

Three single-loaded all-suture anchors (eg, 1.8–2.6 mm) are placed sequentially on the glenoid rim at the chondrolabral junction, approximately 1–2 mm medial to the articular surface, in preparation for LHBT graft attachment. For a right shoulder, anchors are typically placed at the 1 o'clock, 5 o'clock, and 3 o'clock positions; for a left shoulder, anchors are placed at the 11 o'clock, 7 o'clock, and 9 o'clock positions (Fig. 4). Anchor spacing is planned to recreate a continuous bumper. In order to maintain graft tension throughout the fixation sequence, the nonabsorbable suture secured to the cut end is passed out through the posterior viewing portal, and the tension is maintained with the viewing hand. Although the superior anchor is often placed first to capture the tendon near its origin, knot tying is performed starting at the most inferior anchor (5 o'clock) to tension the anteroinferior glenohumeral ligament complex. A flexible/angled drill guide may be helpful for low anchor placement. At each fixation point, anchor sutures are passed through and around the tendon to secure a non-slip fixation point; when capsular advancement is possible, and there is sufficient capsular material for this purpose, the outer suture limb is passed through the adjacent capsule (capsular shift) before being tied around the tendon. During knot tying, the sutures are cyclically tensioned to minimize any subsequent slippage issues.

**Figure 5:**
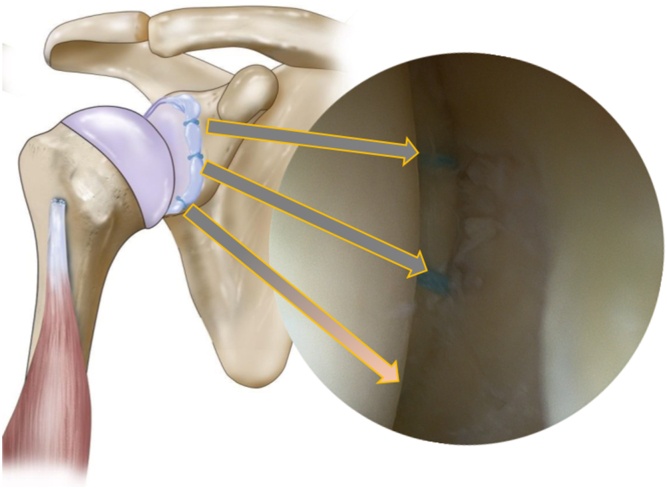
The LHBT autograft is firmly attached to the glenoid rim, the available capsular tissues are attached to the neo-labrum, and the humeral head is reduced into the glenoid by removal of the traction weight. *LHBT*, long head of the biceps tendon.

Once the graft is secured to the glenoid margin, the remaining capsulolabral tissue is advanced and repaired to the neo-labrum using the same anchor sutures to recreate the capsulolabral complex (Fig. 5). Practically, this is accomplished by passing one suture limb through the capsule (capsular shift) before tying it around the tendon graft, with knot tying typically starting inferiorly to tension the anteroinferior glenohumeral ligament.

Occasionally, when additional constraint is desired, supplemental suture anchor fixation points are added in between the 3 primary attachment sites to optimize tendon contact and/or to perform additional capsular plication. The distal end of the graft may be trimmed and removed after it has been secured to the inferior glenoid (at/near the 6 o'clock level) or, when additional inferior/posterior restraint is desired, the excess graft can be incorporated into the inferior capsule or posteroinferior labrum with an additional anchor. After completion of the neo-labrum reconstruction and capsular shift, weight is removed from the traction and the shoulder is reduced. Stability is assessed arthroscopically (restoration of a bumper, centering of the humeral head, and loss of the drive-through sign) and clinically with anterior/posterior load-and-shift testing in neutral and at 90 degrees of abduction with external rotation. This assessment is performed before reconstruction to document baseline instability. If residual translation is greater than acceptable (eg, > grade 1) or persistent engagement is noted, additional capsular plication/rotator interval closure and/or remplissage is performed as indicated.

## Discussion

Shoulder trauma resulting in a labral tear and consequent recurrent instability is often recalcitrant to nonoperative management.^19^ Many factors contribute to successful glenoid labrum repair. The severity of the pathology, patient age and health, and the surgeon's experience must be considered when deciding whether to perform an arthroscopic or open procedure. Labral repair surgeries, such as the Bankart repair, can restore stability while often diminishing the patient's range of motion due to capsular tightening.^24^

The situation is more complex when the labral and capsular tissues are traumatized to such an extent that reattaching them is impossible due to chronicity or structurally futile due to tissue maceration or previous failed surgical interventions.^7^ Currently, these complex situations are popularly treated with the Latarjet procedure, despite a normal or minimally deficient glenoid bony structure.^10^^,^^20^ A more recent addition to the surgical options for this scenario is the DAS procedure, which replicates some aspects of the bony coracoid and short head biceps transfer of the Latarjet procedure with the LHBT.^18^ Both of these latter procedures route the graft through the subscapularis middle/inferior third junction.

The GALIN technique was developed to recreate the labral “bumper” structure and restore the concavity of the glenoid articulation, with the goal of improving resistance to subluxation/dislocation while preserving range of motion. We first described this procedure in a cadaveric biomechanical evaluation of LHBT-based glenoid labrum reconstruction. In that model, the peak force to displacement after reconstruction (16.67 N) was significantly greater than both the labral-deficient state (11.78 N) and the labral-intact state (14.06 N), representing a 41.5% increase vs. the deficient state and an 18.6% increase vs. the intact state (*P* = .011 and *P* < .001). Notably, these differences remained significant despite removal of the capsule and rotator cuff tendons, isolating the stabilizing contribution of the reconstructed labral “bumper.”^27^ More recently, an independent robotic cadaveric study evaluating an LHBT circumferential onlay labral reconstruction similarly demonstrated restoration of key anteroinferior stability metrics (force ratio and lateral humeral head translation) to values not significantly different from the native state in a model without glenoid bone loss, providing additional biomechanical support for LHBT-based neo-labrum reconstruction concepts.^9^

Additionally, the extent of the injury and viability of the remaining static stabilizing structures may restrict which repairs can be predictably employed. Perhaps most pertinent, however, is the amount of glenoid bone loss present in the affected joint. As glenoid bone loss increases, isolated soft tissue stabilization becomes less reliable, and clinical decision-making increasingly shifts toward augmentation strategies (eg, Bankart-based repair with adjuncts) or bony reconstruction procedures (eg, Latarjet/Bristow). While historical thresholds have commonly cited higher levels of bone loss as “critical,” more recent evidence suggests that clinically meaningful bone loss may occur at lower magnitudes, with worse functional outcomes reported above approximately 13.5% and recurrent instability risk increasing with bone loss >15%.^8^^,^^23^ This is best supported by contemporary clinical data from analogous soft tissue augmentation. In a multicenter study of 226 patients, with glenoid bone loss <15%, recurrence after arthroscopic subscapularis augmentation and Bankart repair with reemplisage were 7.0% and 6.1%, respectively. Both displayed excellent Rowe scores in 89.2% and 79.9% of patients, respectively. Therefore, we consider GALIN in carefully selected patients with subcritical bone loss and irreparable capsulolabral tissue, recognizing that bony augmentation remains the preferred choice when bone loss is greater.^16^ From a clinical perspective, while we generally reserve the GALIN technique for patients with <15% glenoid bone loss, the exact glenoid bone loss is difficult to assess (pre-operatively and intraoperatively), and the maximum amount of bone loss that may be present without compromising the results of the GALIN technique has yet to be determined. The exact glenoid bone loss threshold should be explored further to establish reliable benchmarks.

This report focuses on using the patient's own ipsilateral LHBT as a local autograft for neo-labrum reconstruction. Using the ipsilateral LHBT avoids remote-site autograft harvest and associated morbidity and avoids graft-related concerns associated with allograft tissue (eg, cost, availability, storage). In addition, maintaining the proximal biceps attachment may preserve biologic continuity to the graft, although this requires further clinical study.

While the GALIN technique is intended to address shoulders with severe/complete loss of the labral tissue (particularly in the setting of minimal or subcritical glenoid bone loss), its purpose should not be mistaken as a replacement for established stabilization and reconstruction techniques. Several authors have described LHBT-based labral reconstruction/augmentation constructs, including free biceps autograft reconstruction, circumferential onlay constructs (anterior labral circumferential onlay technique), and in-continuity biceps-based reconstructions (Duru technique).^1^^,^^4^^,^^9^ These techniques share the goal of recreating a labral bumper but differ in graft harvest (free vs. in-continuity), fixation patterns, and whether a subscapularis split or tenodesis is performed. For patients with subcritical bone loss and labral tissue damage preventing a predictable repair of the affected static soft tissue stabilizers, other techniques remain relevant, including subscapular augmentation, capsular plication, rotator interval narrowing, and/or Bankart repair with remplissage when clinically indicated.^12^^,^^17^^,^^25^ In cases of significant anterior glenoid bone loss, bony augmentation procedures (eg, Latarjet, Bristow, bone block reconstruction) remain preferred.^2^^,^^6^^,^^11^^,^^21^^,^^26^

Glenoid labrum reconstruction utilizing the GALIN technique, as with any procedure, has both positive and negative attributes. The LHBT provides a readily available local autograft that can recreate a substantial intra-articular bumper; however, tendon dimensions vary between patients, as graft “overstuffing” or inadequate thickness is possible. Because the technique incorporates biceps tenodesis, patients may experience the usual tenodesis-related risks (eg, groove pain, fixation failure, and cosmetic deformity). Overstability may also be a concern, with a potential risk of post-operative stiffness and an accelerated wear pattern that could contribute to early arthritic changes. Robust medium- and long-term clinical outcome data are limited, and the technique should be considered technically demanding with a learning curve. Future studies should evaluate outcomes, complications, and the performance of GALIN across varying degrees of bone loss and instability patterns.

## Clinical experience

Our clinical experience with this difficult subpopulation over the past 16 years has been positive. Over the past 5 years, we have documented 11 cases using this technique in its current form as described in this surgical technique. Nine out of 11 have been performed within the last 5 years, with only 2 reported failures. More patients have undergone this technique; however, we were limited by documentation purposes to formally disclose. Two patients returned with instability that required further surgery. One sustained a traumatic event at a local amusement water park approximately 3 months after the operation, and 1 patient with Ehlers–Danlos syndrome with generalized hyperlaxity required further stabilization. These preliminary observations should be interpreted with caution given the small sample size and relative rarity of this patient demographic, underscoring the need for additional clinical outcome studies.

## Conclusion

Labral deficiency in the setting of recurrent instability remains a challenging problem. Several techniques, including LHBT-based labral reconstruction constructs, have been described; however, standardized indications and clinical outcome data remain limited.^1^^,^^4^^,^^9^ The GALIN technique leverages the ipsilateral LHBT to reconstruct a “neo-labrum” while performing a suprapectoral biceps tenodesis through an all-arthroscopic approach. This technique may be considered for carefully selected patients with recurrent instability, irreparable capsulolabral tissue, and subcritical glenoid bone loss <15%. Further clinical studies are needed to define outcomes, complications, and appropriate bone-loss thresholds.

## Disclaimers:

Funding: No funding was received for the preparation of this manuscript.

Conflicts of interest: The authors, their immediate families, and any research foundations with which they are affiliated have not received any financial payments or other benefits from any commercial entity related to the subject of this article.

Patient consent: Patient consent was not applicable, as this manuscript describes a surgical technique and does not present identifiable patient information, individual patient-level data, or images that could permit patient identification.
